# Preaching to the choir or composing new verses? Toward a writerly climate literacy in introductory undergraduate biology

**DOI:** 10.1002/ece3.5736

**Published:** 2019-10-28

**Authors:** Meghan A. Duffy, J. W. Hammond, Susan J. Cheng

**Affiliations:** ^1^ Department of Ecology & Evolutionary Biology and Office of Academic Innovation University of Michigan Ann Arbor MI USA; ^2^ School of Education University of Michigan Ann Arbor MI USA; ^3^ Department of Ecology & Evolutionary Biology Department of Biological and Environmental Engineering Cornell University Ithaca NY USA; ^4^ Center for Research on Learning and Teaching University of Michigan Ann Arbor MI USA

**Keywords:** climate denial, climate literacy, discipline‐based education research, pedagogy, solastalgia, student affect

## Abstract

Climate change is one of the most pressing issues facing society today, yet a wide range of misconceptions exist in society about whether or why climate change is happening, what its consequences are, and what can be done to address it. Large introductory biology courses present an opportunity to teach a large number of students—some of whom may never take another course focused on climate, ecology, or the environment—about climate change. However, content knowledge alone may not be enough to prepare students to transform their knowledge into action. To begin understanding how content knowledge interacts with student constructions of climate change solutions, we administered and quantitatively analyzed a survey that examined student views of climate change and how they shifted with instruction during an undergraduate introductory biology course at a large Midwestern university. Almost all participants entered the course agreeing that climate change is occurring, and their certainty about the science of climate change increased after instruction. After taking the course, more participants described climate change as having more immediate impacts, reporting that climate change is already harming people and that climate change will harm them personally. However, both at the beginning and end of the course, participants tended to think that humans would either be unable or unwilling to reduce climate change. They were also more worried about climate change at the end of the course than they were before. Increased concern might result from students becoming more certain of the science and severity of climate change, while remaining pessimistic that humans will effectively act on climate change. This pattern suggests instructors have opportunities to modify curricula in ways that leave students with a greater sense of empowerment and efficacy; we suggest questions that instructors can ask themselves in order to modify their courses with this goal in mind.

## Graphical abstract

We found that almost all students arrived in an introductory biology course accepting climate change, and, after instruction, they become more certain that climate change is occurring. However, students both entered and left the course pessimistic about whether humans will successfully address climate change and became more worried about climate change after instruction. We argue that meaningfully increasing students' climate literacy requires not only increasing student knowledge, but also preparing and inspiring them to make use of that knowledge.

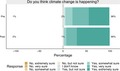

## 1 INTRODUCTION

I understand that global warming is an important part of learning from this class, but at some point one of the lectures gave me an anxiety attack, so that wasn't fun.Anonymous Introductory Biology Student

Climate change is one of the most pressing issues facing society: It is a world‐altering phenomenon that has, and will continue to have, noticeable impacts on the lives of today's college students (IPCC, 2018; USGCRP, 2018). Fortunately, introductory science courses, including introductory biology courses, offer opportunities to teach a large audience of students with a wide range of interests, backgrounds, and experiences regarding climate change. Instructors in these courses typically aim to increase students' knowledge of content, but as the student quoted in the epigraph above makes clear, instruction can affect more than content knowledge. This anonymous student comment, left as course evaluation feedback for the large undergraduate biology course examined in this article, reminds us that instruction can also influence how students feel, and consequently, how students engage with and use content knowledge—or not. The challenge confronting many instructors is this: How can we ensure that students leave our courses not only with an increased understanding of the science of climate change (including the potential severity of its impacts), but also with a sense of purpose and empowerment?

Taking up this question requires us to revisit what we mean by “climate literacy,” and to carefully consider what kind of climate literacy we hope students achieve in introductory science courses. As characterized by the United States (US) Global Change Research Program (2009), the “climate‐literate person” possesses four literate features: She comprehends the core aspects of the planet's climate system; is ready to appraise climate‐related information and make judgments about its credibility; meaningfully communicates about climate as well as climate change; and is prepared to respond appropriately to the realities of climate change (p. 4). This way of thinking about climate literacy reserves an important place for knowledge about climate, but it does not stop there. The “literacy” part of climate literacy encompasses comprehension, but also extends to a critical capacity to assess new information, and to responsively and responsibly intervene in the world, acting on—and in response to—knowledge about climate. After all, when we talk about “literacy” in other contexts, this word designates not just the ability to *read* and process content, but also to *write*—acting on, extending, and recomposing information. A higher level of climate literacy might therefore be described as students understanding the causes and consequences of climate change *and* knowing how to use and build on that knowledge—something we could metaphorically call a *writerly* stance toward climate science.

Nevertheless, research on climate education often focuses primarily or exclusively on students' knowledge of climate change, attending more closely to student understandings and misunderstandings than to other aspects of climate literacy (e.g., Pascua & Chang, 2015). This narrower, knowledge‐centric approach to climate literacy is one Busch and Román (2017) discuss in the K‐12 educational context as “derived” climate science literacy, which they contrast with “fundamental” climate science literacy: an “ability to read, evaluate, and produce” climate‐related scientific content, making scientific knowledge intelligible and actionable (Busch & Román, 2017, p. 121; see also Norris & Phillips, 2003; Yore, Bisanz, & Hand, 2003). Developing alongside this attention to both derived and fundamental scientific literacies, a growing body of scholarship has identified knowledge of social systems, actors, and practices as important aspects of scientific literacy, generally (e.g., Hand, Lawrence, & Yore, 1999; Hurd, 1998), and climate science literacy, specifically (e.g., González‐Gaudiano & Meira‐Cartea, 2010; Shwom, Isenhour, Jordan, McCright, & Robinson, 2017). Hurd (1998), for instance, has characterized “[b]ehaviors associated with the production and utilization of science knowledge in human affairs” as “represent[ing] the civic basis of scientific literacy” (p. 413). For their parts, Shwom et al. (2017) have charged that while faulty models of the climate system impede climate literacy, “faulty models about social, political, and economic systems are just as damaging to climate literacy, given that they can foster misunderstanding about how those systems contribute to climate change as well as possibilities for mitigation and adaptation” (p. 377).

We propose that these more critical, socially conscious, and agentic approaches to scientific literacy are models of *writerly climate literacy*, a term we use to denote not just a cognitive readiness to interpret, assess, and write scientific content, but also a strategic, evidence‐backed sense of efficacy toward climate action: a broader affective, social, and intellectual readiness to act on knowledge about climate, translating and recomposing what is learned in the classroom into socially and scientifically conscious action beyond the classroom. Even if our courses impart a climate‐related “functional scientific literacy” (Zeidler & Newton, 2017) that “moves beyond retention of scientific fact” to contemplate the ethical stakes and social consequences of (not) acting on those facts (p. 60), such climate literacy may not amount to much if students *also* are too discouraged, disengaged, or overwhelmed to put it into practice. A sense of efficacy and motivation to act are core components of writerly climate literacy, distinguishing it from more readerly engagements with climate knowledge. If the goal of introductory science coursework is to promote the rich, multidimensional kind of climate literacy outlined by the US Global Change Research Program (2009), there is good reason for science educators to expand their attention from climate knowledge alone to also include students' assumptions and feelings about climate change—and about how to respond to it.

Within the US, scholars have argued that American adults' perceptions of climate change can be grouped into six categories—alarmed, concerned, cautious, disengaged, doubtful, and dismissive (Maibach, Roser‐Renouf, & Leiserowitz, 2009)—and, relatedly, that the best approach to communicating climate change with the public will vary with each of these groups (Roser‐Renouf, Stenhouse, Rolfe‐Redding, Maibach, & Leiserowitz, 2015). Similarly, when designing curricula and enacting pedagogy, it is important to remember that students are not merely “empty ‘mind[s]’ passively open to the reception of deposits” (Freire, 2018, p. 75). Instead, students arrive in courses with prior knowledges, beliefs, conceptual frameworks, and misconceptions that influence their engagements with course materials, and affect whether and how they learn course content (see, e.g., Aksit, McNeal, Gold, Libarkin, & Harris, 2018; Chi, 2005; Gilbert & Watts, 1983; Rissler, Duncan, & Caruso, 2014). Students' classroom engagements with climate change are no exception to this rule (Dupigny‐Giroux, 2010; Lombardi & Sinatra, 2012). Previous research on college students has found that students generally agree that climate change is happening, but that they also harbor misconceptions regarding climate change (see, e.g., Pfautsch & Gray, 2017; Wachholz, Artz, & Chene, 2014). Fortunately, a study of 17 college students who took a series of sustainability‐related courses found that instruction can improve climate literacy and certainty regarding climate change, while also increasing students' sense of urgency regarding climate change (Burkholder, Devereaux, Grady, Solitro, & Mooney, 2017).

Even so, increased information related to climate change may, on its own, not cause significant changes in behavior or equip students to successfully combat what Gifford (2011) calls “the dragons of inaction” (see, e.g., Corner et al., 2015; González‐Gaudiano & Meira‐Cartea, 2010; Wachholz et al., 2014; Zeidler & Newton, 2017). Apparently, there is a disconnect separating student certainty and concern about climate change from readiness to act in response to that certainty and concern. Exacerbating matters, students may feel fearful, sad, remorseful, despairing, and angry in response to climate change (Pfautsch & Gray, 2017) and become more worried after instruction (Holthuis, Lotan, Saltzman, Mastrandrea, & Wild, 2014). This affective impact of climate pedagogy is potentially dangerous, if it means that students leave a course feeling that there is nothing they can do to combat climate change. After all, a constructive sense that one can make a difference can be an important factor in whether students adopt pro‐environmental behavior (Ojala, 2012, 2015) and an absence of self‐efficacy can short‐circuit climate action (Corner et al., 2015). In this way, increasing students' sense of urgency about climate change may have negative, unintended consequences, if this sense of urgency is not buoyed by and balanced with a sense of efficacy where climate action across scales is concerned. Instructors might be able to counter this potential negative consequence of instruction if they reimagine the classroom as a place where students not only gain knowledge about climate change and its impacts on different communities, but also gain knowledge about how to use that knowledge and leave feeling empowered to do so.

Taken together, these prior studies suggest that the problem facing science educators is not merely whether or not students accept that climate change is happening, but also whether students have clear and accurate understandings of why climate change is happening, and—crucially—whether students leave their courses confident that their understandings can be put to productive use in some way. Putting it differently: Are our classrooms preparing students only for a “readerly” climate literacy, where they take in information about climate, without corresponding attention to more participatory “writerly” forms of climate literacy, where they feel prepared to take action in response to what they have learned? If students come to our courses already accepting climate change is occurring, are we merely “preaching to the choir,” so to speak, when we have the opportunity to support students in composing new verses?

To help answer these questions, our study focused on students' constructions of climate change in an introductory biology course at a large Midwestern university in the US. We sought to characterize student constructions of climate change in a number of ways. First, we were interested in better understanding whether these students accepted climate change, and whether they understood its impacts and the scientific consensus regarding climate change when they entered the course—as well as how those views and understandings changed after instruction. Second, we were interested in student affect—especially how worried students were and how important climate change was to them personally—and how these sentiments shifted over the semester. Finally, we sought to understand what students thought about the potential to act to slow or stop climate change, and whether those thoughts changed after instruction. Our study adds to a rapidly growing body of literature on student understandings of climate change and suggests ways instructors can modify their teaching to expand beyond a readerly climate literacy. By better addressing the impact of learning about climate change on student affect, we can move toward a more writerly form of literacy where students are prepared to respond to the realities of climate change.

## 2 METHODS

**Figure 1:**
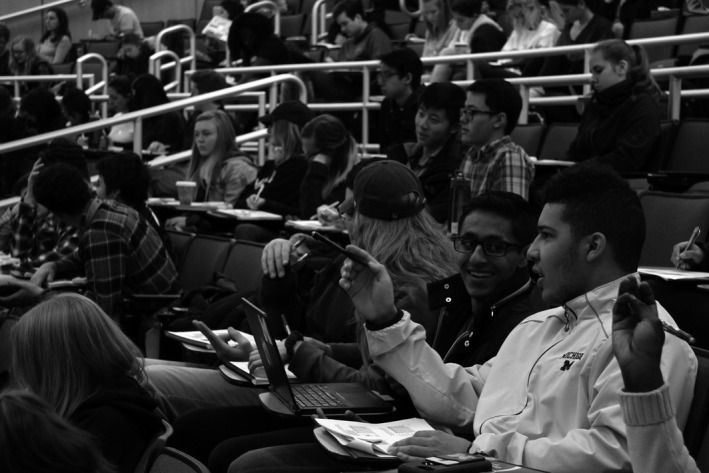
Most students in the introductory biology course we surveyed attended a large professor‐led lecture section, similar to the one depicted in this photograph. Photograph credit: Martin Springborg

This study was conducted in an introductory biology course geared toward science majors at a large Midwestern university during two semesters in the 2017–2018 academic year. Most students in this course attended (in‐person) a large lecture section led by a professor (Figure 1) and a smaller discussion section led by a teaching assistant; a smaller subset of the students attended (also in‐person) a smaller lecture and discussion led by a lecturer. The instructors of the large lecture section, and some of the course lectures and discussion section materials, differed between the two semesters, but the climate change module was largely consistent, including having the same learning objectives. The climate change course materials were based in part on materials developed by the American Association for the Advancement of Science (AAAS, 2014). In particular, the instructors sought to highlight five points: (a) Climate change is happening now, (b) Climate change is largely caused by humans, (c) The best way to slow climate change is to reduce greenhouse gas emissions, (d) Climate change is already altering species and ecosystems, and (e) Climate change affects human health. Climate change was the primary focus of one 80‐min lecture (out of 24 lectures total) and one 80‐min discussion section (out of 10 discussions total); climate change was also covered in additional lectures (e.g., discussing the spread of Lyme disease in the Midwest during a lecture on emerging infectious diseases).

Using a university‐designed online system, we invited students enrolled in this course to participate in surveys that asked them to describe their perceptions of climate change. In both semesters, students received an invitation to take the survey before the course module on climate change and again after the course module was completed. In the first semester, the premodule survey invitation was sent mid‐semester and the postmodule survey invitation was sent near the end of the semester. In the second semester, invitations were sent at the beginning of the semester and again at the end of the semester. Students who completed the premodule or postmodule surveys received extra credit.

The surveys consisted of Likert‐scale and open‐ended questions, some of which were based on questions from the Pew Research Center (2016) survey and the Climate Change in the American Mind survey (Leiserowitz, Maibach, Roser‐Renouf, Feinberg, & Rosenthal, 2016), iterations of which have provided a basis for other climate science education studies (e.g., Holthuis et al., 2014). In response to a lower‐than‐expected response rate in 2017 (possibly from survey fatigue), we administered a shorter survey in 2018. In this paper, we focus on the student responses to the Likert‐scale questions. In 2017, the premodule survey contained 15 Likert‐scale questions and a validation question, and the postmodule survey contained 16 Likert‐scale questions and a validation question. The 2018 survey shared 7 (beginning of semester) or 8 (end of semester) identical Likert‐scale questions with the 2017 survey. In 2018, the validation question was inadvertently omitted from the premodule survey, but was on the end‐of‐semester survey. The full surveys are provided in the Appendices S2 and S3.

A total of 581 students were enrolled in the first semester and a total of 588 students in the second semester. In our analysis, we only included responses from students who completed both the premodule and postmodule surveys and answered validation questions correctly. With these criteria, we had a 21% response rate (*n* = 120) in the 2017 semester and a 45% response rate (*n* = 263) in the 2018 semester. Participants were predominately white (72% in 2017, 59% in 2018), women (78% in both semesters), first or second year undergraduates (83% in 2017, 80% in 2018), and from a household with an annual income above $100,000 (50% in 2017, 47% in 2018). In 2017, students in the course had 1.0 previous Biology credit taken at the university, on average: 384 of the 581 students had not taken any previous Biology credits at the university; 96 had taken 4.0 previous Biology credits. In 2018, the average was 1.8 credits: 350 of the 588 students had not taken any previous Biology credits at the university; 203 students had taken 4.0 previous Biology credits. Additional participant demographic data from both semesters are given in Table 1. For survey questions that were identical in both semesters, data from these semesters were combined for visualization and analyses. Because some questions were given in both semesters but others only in 2017, the figures indicate the number of respondents; questions with approximately 380 respondents were those given in both semesters. Data were analyzed using R v. 3.5.2. For many questions, we report only summary statistics (e.g., the percent of students who selected a particular response category); for some questions, we also used proportion tests to compare the change in the proportion of students choosing a particular response at the beginning of the semester versus the end of the semester. Data and code are available at https://doi.org/10.5281/zenodo.3334292.

**Table 1:** Demographics of survey respondents from university‐provided data

Variable	Category	Semester 2017		Semester 2018	
		*n*	%	*n*	%
Gender	Female	94	78	205	78
	Male	26	22	58	22
Race	American Indian	0	0	1	0.4
	American Indian, Asian, Pacific Islander, White	1	1	0	0
	Asian	17	14	53	20
	Asian, Hispanic, White	0	0	1	0.4
	Asian, White	2	2	6	2
	Black	3	3	14	5
	Black, Hispanic	0	0	1	0.4
	Black, White	1	1	1	0.4
	Hispanic	2	2	6	2
	Hispanic, White	4	3	13	5
	White	86	72	154	59
	Prefer not to say	4	3	13	5
Geography	In‐state student	84	70	181	69
	Out‐of‐state student	36	30	82	31
Class year	First year	61	51	90	34
	Sophomore	38	32	120	46
	Junior	15	13	41	16
	Senior	5	4	11	4
	Not candidate for a degree	1	1	1	0.4
Household income	>$200,000	31	26	61	23
	$150,000–$200,000	8	7	27	10
	$100,000–$150,000	20	17	37	14
	$75,000–$100,000	9	8	18	7
	$50,000–$75,000	9	8	9	3
	$25,000–$50,000	9	8	31	12
	<$25,000	7	6	24	9
	Don't know	3	3	4	2
	No response	24	20	52	20

Note

Survey respondents include individuals who submitted the premodule and postmodule survey and answered the validation question(s) on the surveys correctly (*n* = 120 in semester 2017, *n* = 263 in semester 2018). The number of enrolled students was 581 in semester 2017 and 588 in semester 2018. The class was 70% female in 2017 and 69% in 2018. Thus, the female skew in the responses likely results, in part, from the demographic makeup of the class.

## 3 RESULTS

### 3.1 Student conceptions of climate change science and scientists

**Figure 2:**
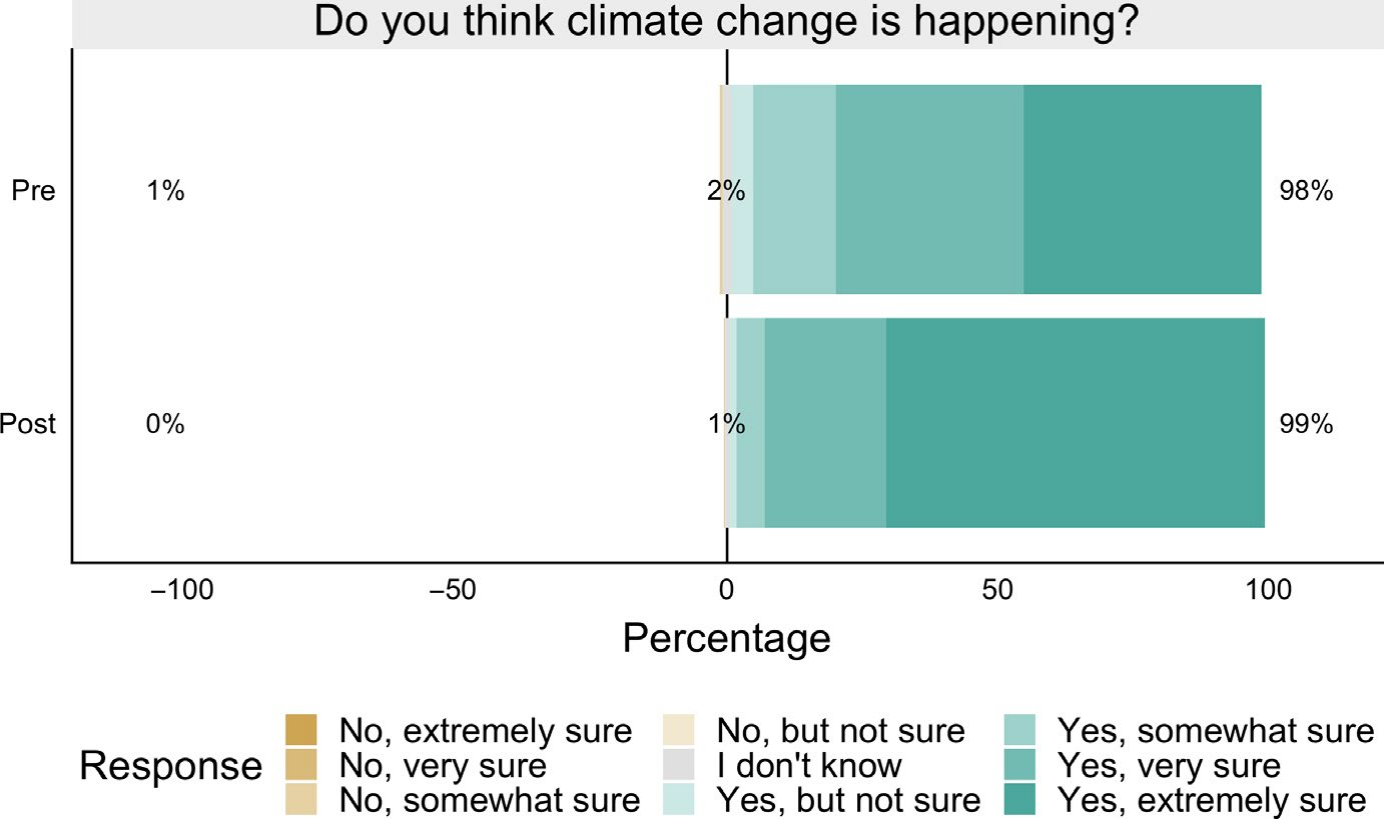
Student responses (combined from 2017 and 2018) to the question, “Do you think that climate change is happening?” when asked at the beginning (top row) and end (bottom row) of the semester. At the beginning of the semester, 98% of participants chose one of the “yes” options (plotted in shades of green and to the right of the vertical center line), 2% chose “I don't know” (plotted in gray in the center of the plot), and 1% chose one of the “no” options (plotted in shades of brown on the left side of the plot). Most of these students arrived in the course already accepting that climate change is occurring and after instruction, more of them were extremely sure climate change is occurring. Only 44% were extremely sure that climate change is happening (darkest green fill) at the beginning of the semester, compared to 70% at the end of the semester

Participants entered the course already accepting that climate change is occurring and more of them felt extremely sure of this fact by the end of the semester (Figure 2). Ninety‐eight percent reported that they thought climate change is occurring prior to instruction; 99% reported this thinking at the end of the course. The percentage of these students who were “extremely sure” that climate change is happening increased from 44% at the beginning of the semester to 70% at the end of the semester (*χ* ^2^ = 56.2, *p* < .0001).

**Figure 3:**
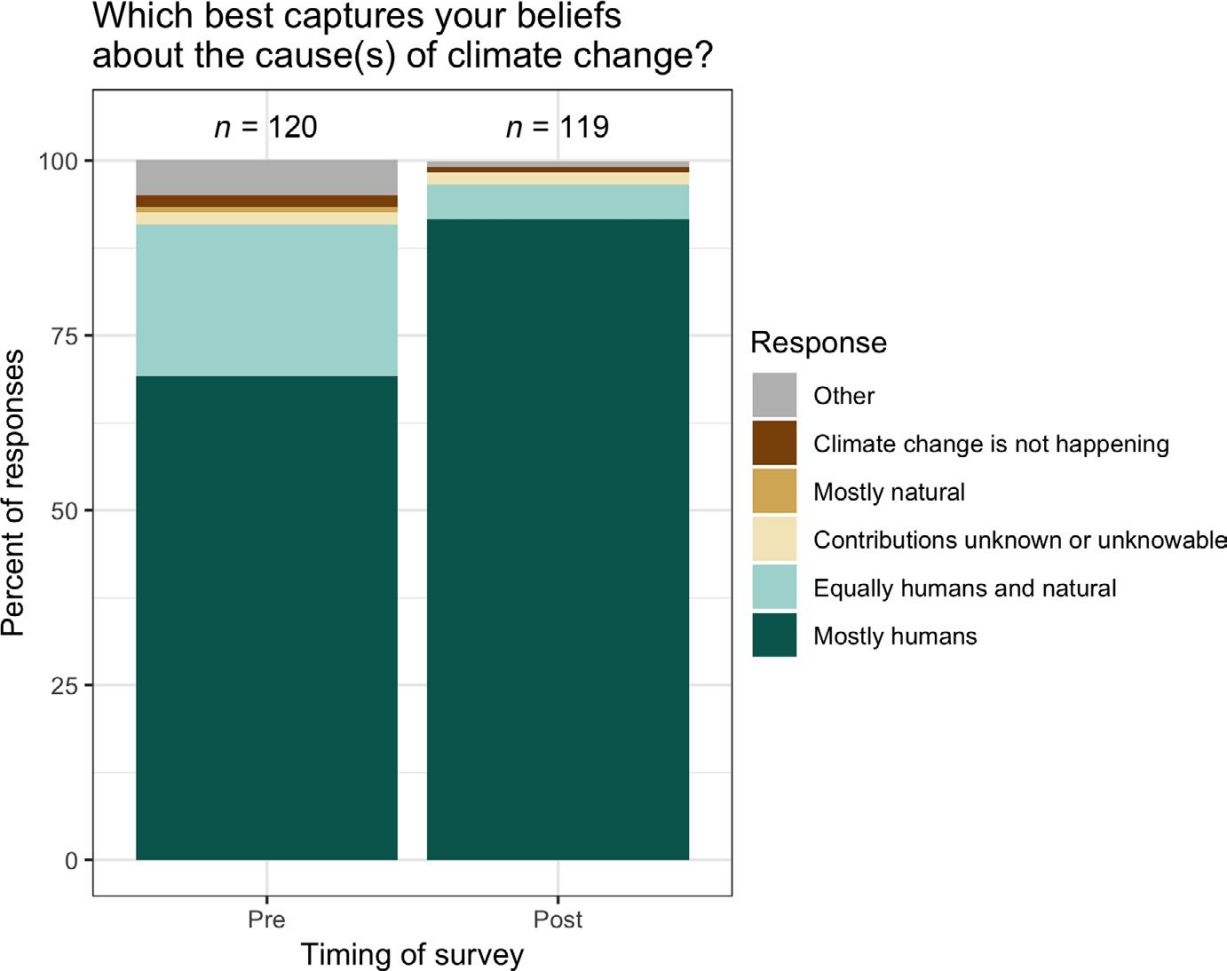
Student responses from 2017 to the question: “Which of the following best captures your beliefs about [the cause(s) of] climate change?” Most participants recognized that humans are primarily responsible for climate change at the beginning of the semester, but substantial numbers thought that climate change is caused equally by human activities and natural changes in the environment. At the end of the semester, 92% identified that climate change is caused mostly by human activities. This question was not asked in 2018 (see Section 2)

The percentage of participants who correctly identified humans as the primary cause of climate change significantly increased from 69% at the beginning of the semester to 92% after the climate change module (*χ* ^2^ = 17.6, *p* < .0001; Figure 3). This shift was associated with a decrease in the percentage who described climate change as equally caused by humans and natural processes (from 22% to 5%; *χ* ^2^ = 12.8, *p* < .001; Figure 3). Both of these shifts indicate that participants more accurately described the causes of climate change after taking the course's climate change module.

**Figure 4:**
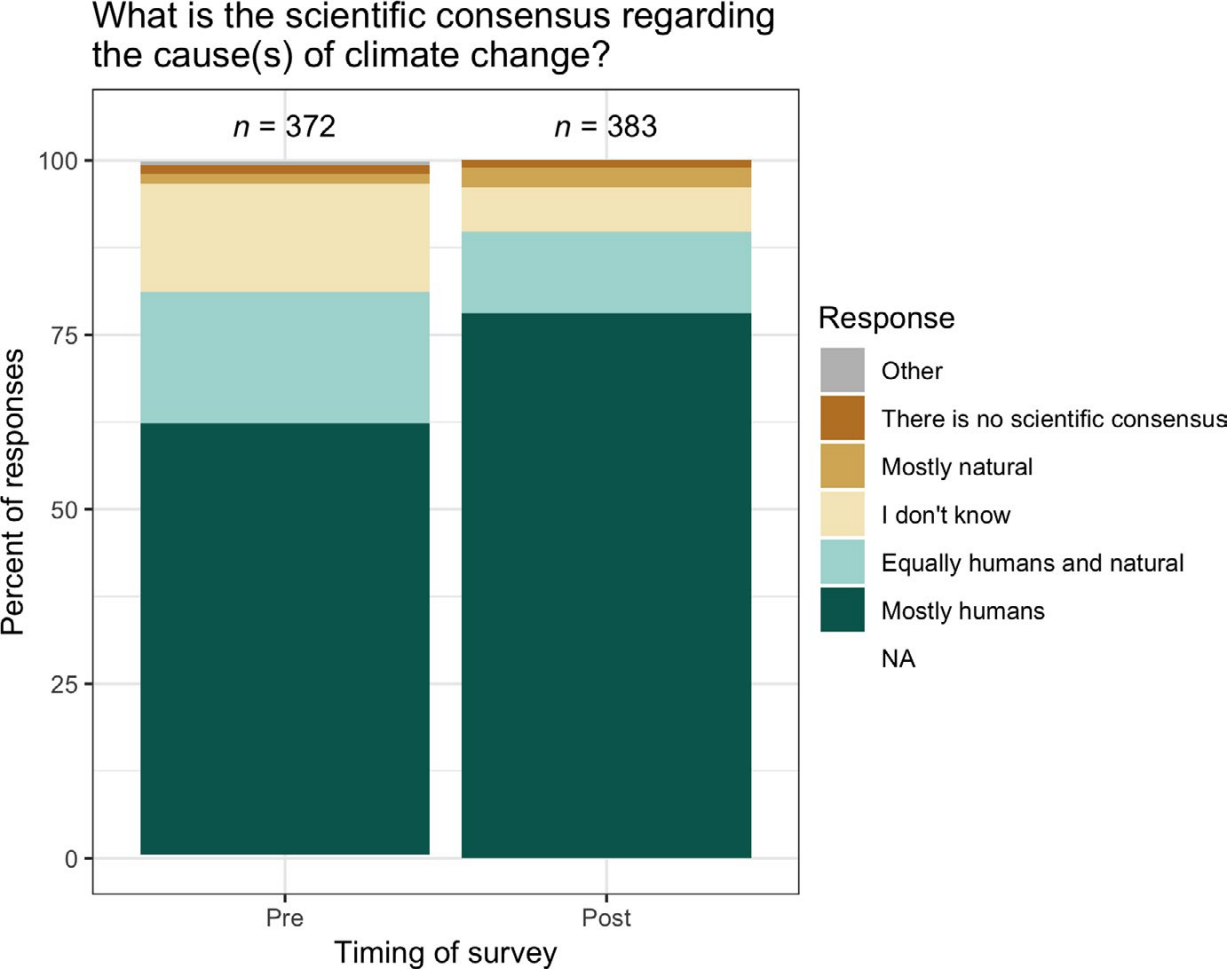
Student responses (combined from 2017 and 2018) to the survey item: “The scientific consensus is that climate change is….” At the beginning of the semesters, most participants (62%) correctly identified that the scientific consensus is that climate change is caused mostly by humans, but a large number (19%) nevertheless incorrectly reported that the consensus is that human activities and natural processes are equally responsible. By the end of the semester, 78% correctly identified that the scientific consensus is that climate change is mostly caused by humans, while only 12% incorrectly thought that humans and natural causes contribute equally to climate change

**Figure 5:**
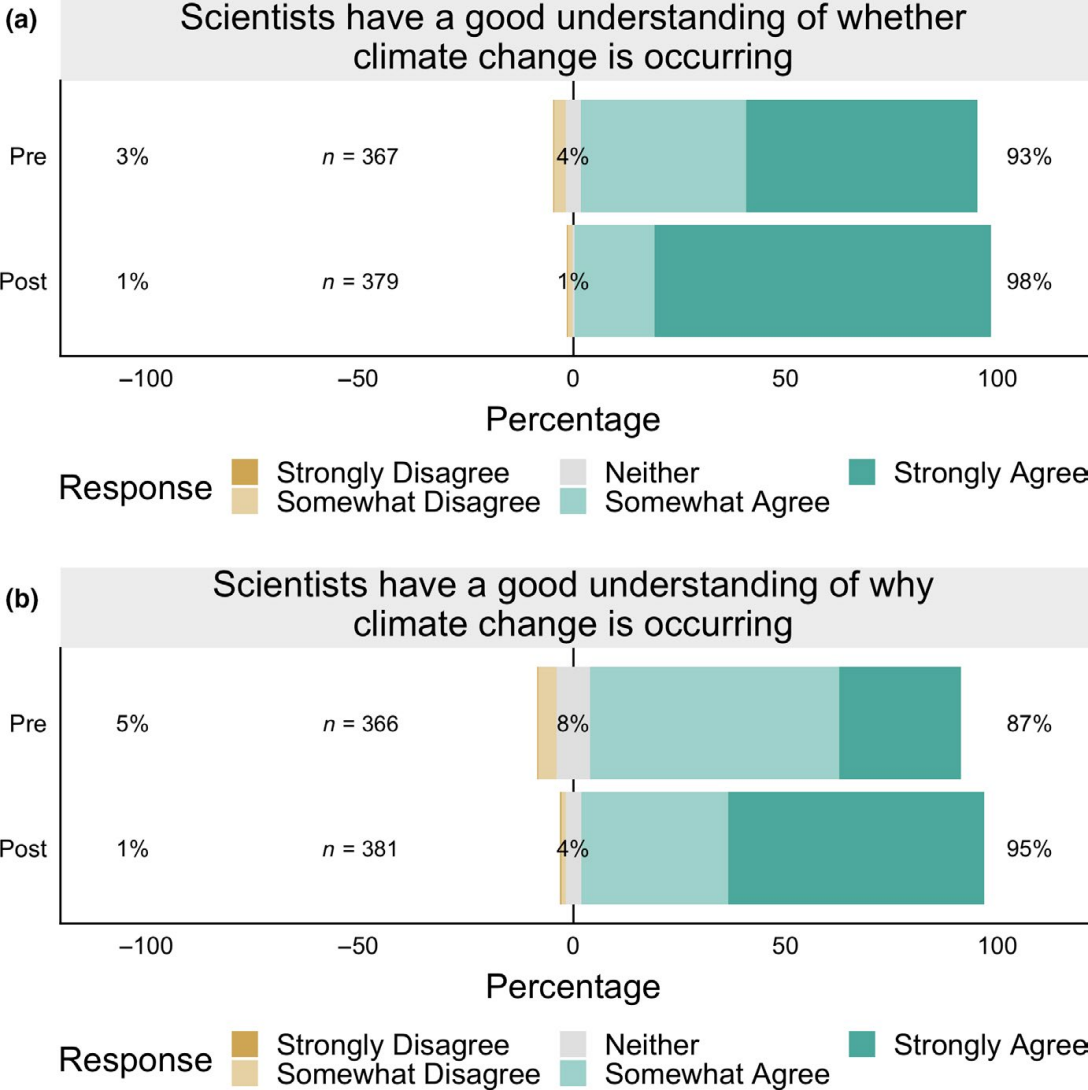
Student Likert‐scale responses (combined from 2017 and 2018 semesters) to the statements (a) “Scientists have a good understanding of whether climate change is occurring” and (b) “Scientists have a good understanding of why climate change is occurring.” Participants agreed that scientists understand whether and why climate change is occurring, and shifted to holding those views more strongly by the end of the semester (Note: Students who chose “I don't know” [1–6 students per semester‐question combination] are not plotted, which explains the slight differences in percentages between the results given in the text and on the figure; the numbers of student responses included in the plots are indicated on the figure)

Participants also more accurately described the scientific consensus around the cause(s) of climate change after the course's climate change module. Although these students tended to enter the course already correctly describing that there is a scientific consensus that humans are the primary drivers of climate change, the proportion of them responding this way significantly increased by the end of the semester (from 62% to 78%; *χ* ^2^ = 22.9, *p* < .0001; Figure 4). The percentage agreeing that scientists have a good understanding of *whether* climate change is occurring increased from 92% to 98% (Figure 5a; note that the figure omits students who chose “I don't know,” which explains the slight difference in percentages), and participants shifted to holding these views more strongly (54% “strongly agreed” prior to instruction vs. 79% at the end of the semester). Moreover, by the end of the course, more agreed that scientists understand *why* climate change is occurring (an increase from 86% to 94%, Figure 5b), and these students also shifted to holding these views more strongly (28% “strongly agreed” prior to instruction vs. 60% at the end of the semester).

### 3.2 Student personalization of climate change impacts

**Figure 6:**
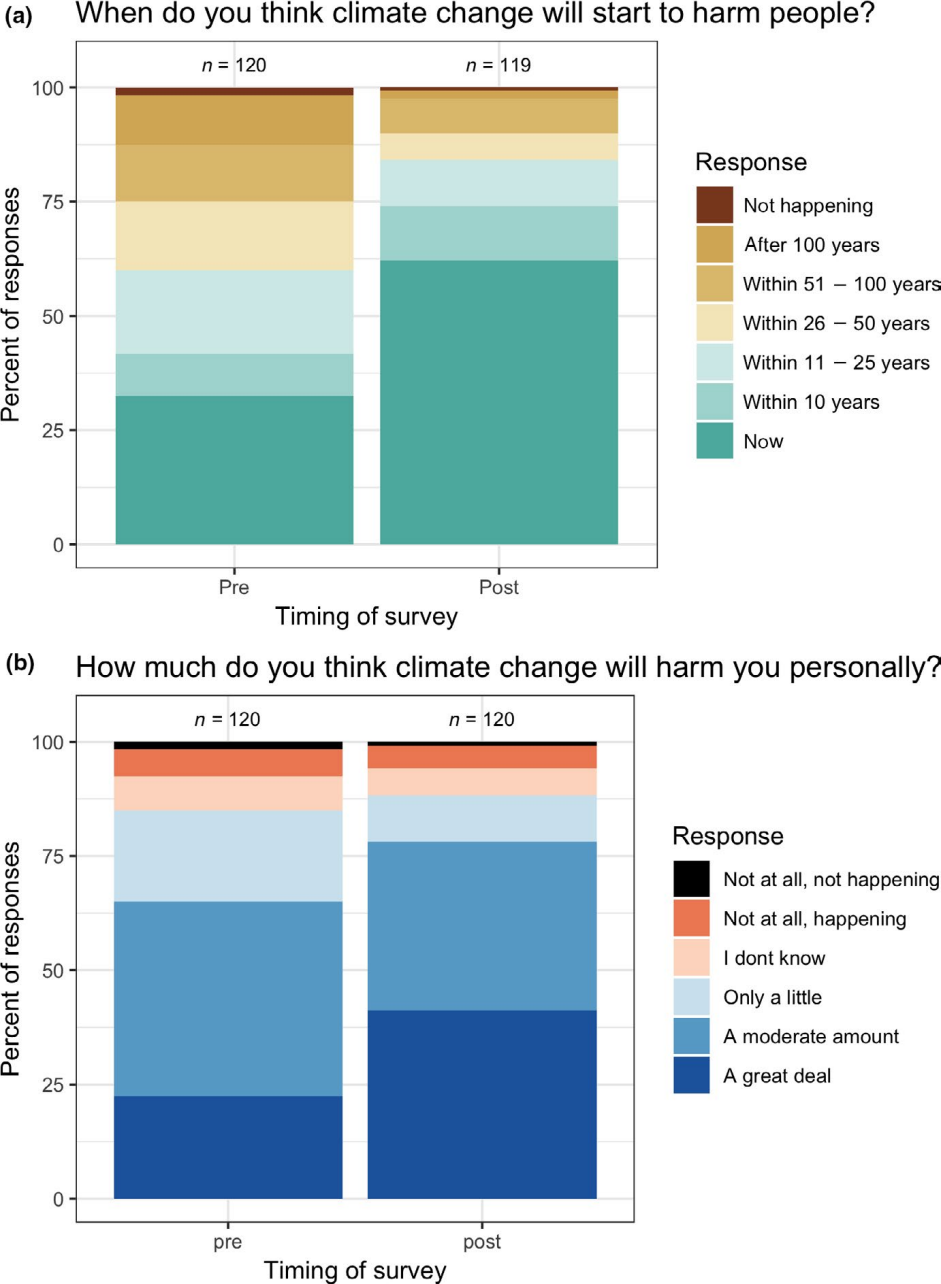
(a) Student responses in 2017 to the question: “When do you think climate change will start to harm people?” When asked at the beginning of the semester, only 33% of participants recognized that climate change is already harming people now, whereas at the end of the semester 66% recognized this. (b) Student responses in 2017 to the question: “How much do you think climate change will harm you personally?” The most common response at the beginning of the semester was “a moderate amount,” compared with “a great deal” at the end of the semester. The impacts of climate change came to be seen as more immediate and more personal by the end of the semester. These questions were not asked in 2018 (see Section 2)

At the end of the course, participants described climate change as having more immediate and more personal impacts compared to those impacts they described at the course's beginning (Figure 6). Before the climate change module, most of them did not recognize that climate change was already harming people: Only 33% chose “now,” while the remainder selected options describing climate change as beginning to harm people anywhere from within 10 years to after 100 years. By the end of the course, the percentage who selected “now” increased to 63% (Figure 6a, *χ* ^2^ = 19.9, *p* < .0001). However, even at the end of the course, 8% still thought climate change would not start to harm people until 51–100 years from now, and 2% thought it would take over 100 years. There was also a significant shift in how participants described the magnitude of the impacts of climate change that they expected to personally experience. Before the climate change module, a plurality of these students (43%) chose “a moderate amount” (Figure 6b). However, by the end of the semester, the most common response to this question was “a great deal” (41%).

### 3.3 Changes in student affect regarding climate change

**Figure 7:**
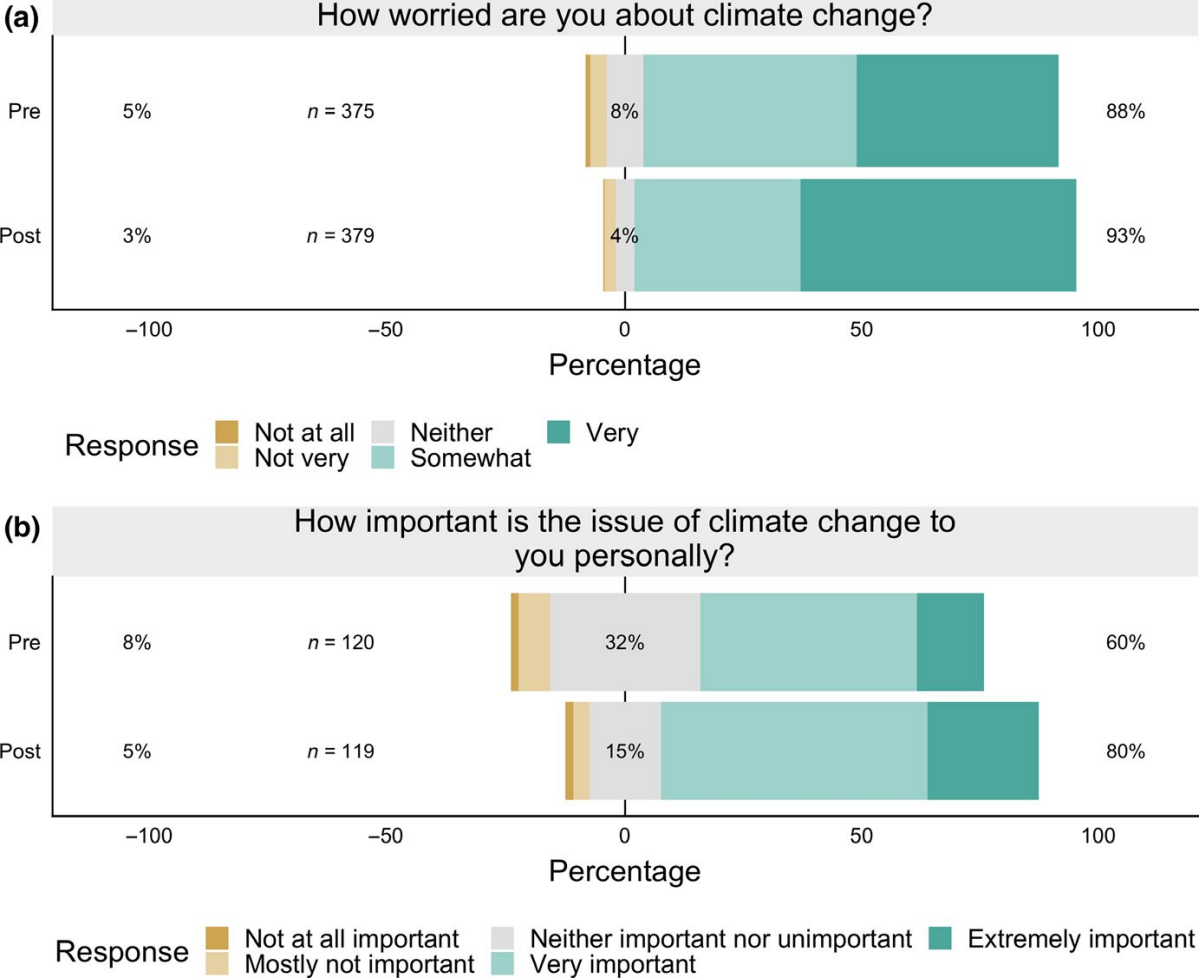
(a) Student responses in 2017 and 2018 to the question: “How worried are you about climate change?” and (b) student responses in 2017 to the question: “How important is the issue of climate change to you personally?” Participants became significantly more worried about climate change by the end of the semester and reported that climate change was more personally important to them

Over the course of the semester, participants became more worried about climate change (Figure 7a). Although the percentage who described being worried about climate change only increased from 88% to 93%, the proportion who were “very” worried increased from 43% to 58% (*χ* ^2^ = 17.8, *p* < .0001). In 2017, students were also asked about how important climate change was to them personally. The percentage of participants who reported that climate change was personally important to them increased from 60% to 80% after the climate change course module (Figure 7b, *χ* ^2^ = 10.2, *p* = .001), and the percentage who thought climate change was an extremely important personal issue increased from 14% to 24%.

**Figure 8:**
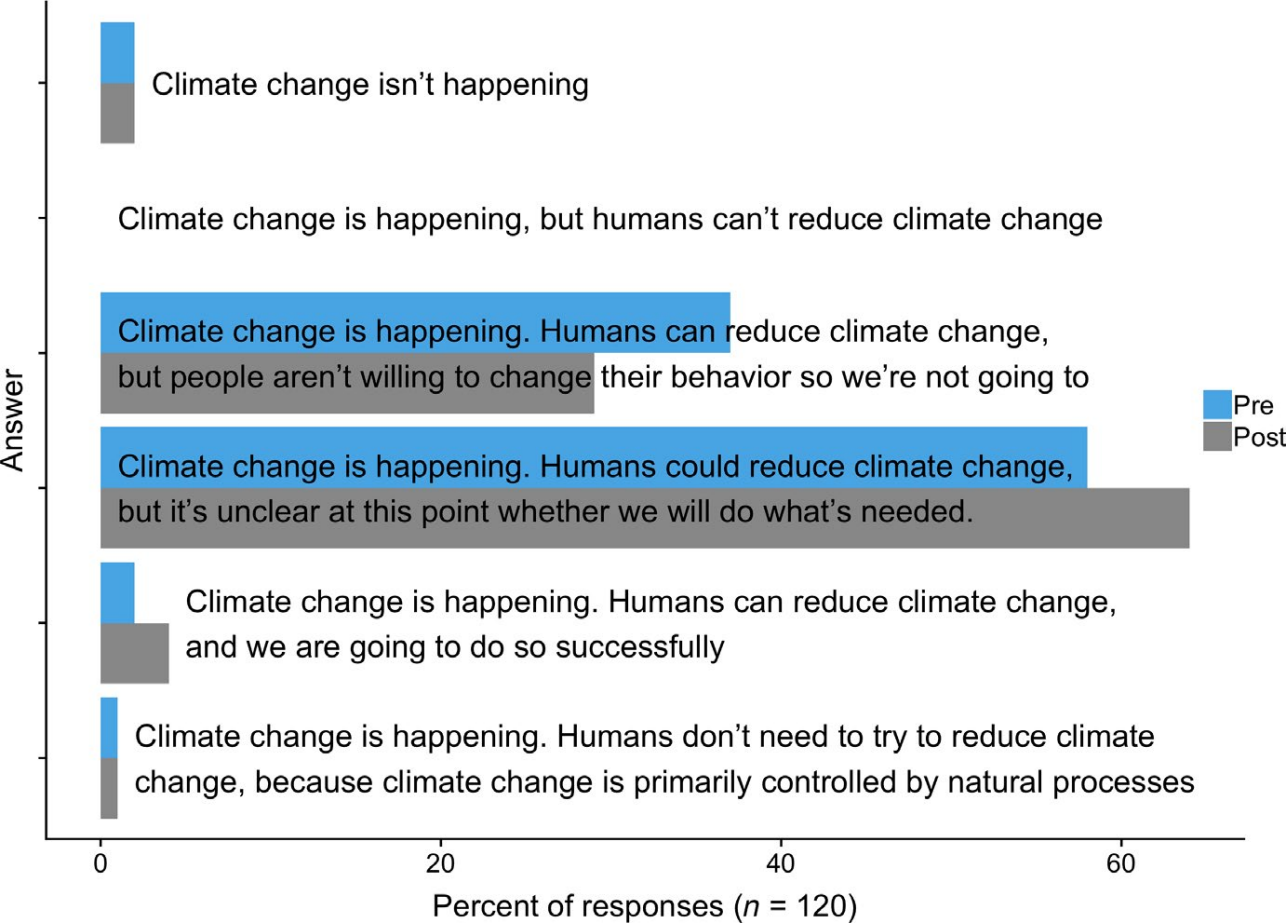
Student responses in 2017 to the question: “Which of the following statements comes closest to your view?” Both at the beginning and end of the semester, participants were not confident that humans would reduce climate change. This question was not asked in 2018 (see Section 2)

However, there was one notable way in which participants' views did not substantially change after the climate change module. Before the course module, 58% reported thinking that humans could reduce climate change but that it was unclear whether they would (Figure 8). The next most common response (37%) was that humans could reduce climate change, but were not willing to change their behavior—thus no meaningful action would be taken. After the course module, participants continued to report uncertainty regarding whether humans would do what is needed to reduce climate change (64%) or else concluded that humans were unwilling to change their behavior (29%)—meaning that, by the course's end, 93% of participants were either unsure whether humans would take meaningful action in response to climate change or were confident that humans would not do so. At the end of the course, only 4% reported thinking that humans would successfully reduce climate change. These results suggest that learning more about the science of climate change—including that humans are the primary cause of climate change—did not have much impact on student views regarding whether humans would address the problem.

## 4 DISCUSSION

Climate change already affects individuals, populations, and ecosystems and will continue altering the lives of today's college students. Do students understand these effects? Are they concerned? And do they think humans can—and will—take action to fight climate change? Our survey indicates that participants entered this course accepting that climate change is occurring, and that their understanding of and concern about climate change increased during the course. Even more notable, perhaps, is what did not change: These students entered and left the course pessimistic about whether humans would successfully address climate change, despite their having the means to do so. As we discuss in this section, meaningfully increasing students' climate literacy requires not only increasing student knowledge, but also preparing and inspiring them to make use of that knowledge. Introductory science coursework can and should support these efforts; our climate future depends, in part, on the kind of climate literacy our pedagogy supports. 

If our participants' responses are any indication, students may be entering postsecondary education in the US with a higher confidence in the realities of climate change than existing surveys might lead us to expect. Compared to surveys of the general US population, the students who participated in our survey arrived in the course with better‐than‐average acceptance and understanding of climate change (Leiserowitz et al., 2018). Before the course module, 98% of these students already agreed that climate change is occurring. Thus, even prior to instruction in this course, participants' responses indicated greater acceptance of climate change than students in a recent survey of American college students—75% acceptance (Wachholz et al., 2014)—and more than the general American public—70% acceptance (Leiserowitz et al., 2018). Study participants were also much more likely than the general American adult population to correctly identify that the scientific consensus is that humans are primarily responsible for climate change: 62% of participants recognized this at the beginning of the course, versus just 27% of US adults in a general survey (Pew Research Center, 2016).

If students enter their science courses already accepting climate change, our courses may often be preaching to the choir—but that does not mean instruction has no effect or value. After instruction, participants became more certain that climate change is happening, that humans are primarily responsible, and that there is a strong scientific consensus that climate change is happening and caused by people—findings broadly consistent with earlier studies (e.g., Holthuis et al., 2014). However, considering that participants already accepted that climate change is happening when they entered the course, class time might be better devoted to more advanced or nuanced topics—topics that take the realities of climate change as their starting premise, and that build on, extend, and add meaningful complexity to student understandings.

Rather than devoting our efforts primarily or exclusively to cultivating a readerly climate literacy in students, the fact that many students come to our courses already accepting climate change means that science educators have a profound opportunity to help students develop a more writerly climate literacy, readying them to act responsively and responsibly on their developing climate change knowledge. Our task as instructors is not to “deposit” knowledge in students' heads (see Freire, 2018), but to support them in intervening in the very systems our courses help students to better understand. Some initial ideas for shifting the focus of climate pedagogy are discussed in greater depth later in this section.

Before discussing these ideas, it is worth noting some additional findings from our study, which point to areas where student understandings shifted. Participating students tended to enter the course thinking that climate change is not yet harming people—a finding consistent with other recent studies (Leiserowitz et al., 2018; Spence, Poortinga, & Pidgeon, 2012). At the beginning of the course, only 33% of participants correctly identified that climate change is already harming people. Instead, at the beginning of the semester, these students thought climate change would not harm humans for at least another decade, and a quarter of them thought it would be at least 50 years before climate change would start to harm people. The number who initially recognized that climate change is already harming people was surprisingly low, especially given these students' overall acceptance of climate change (see above). After all, existing research suggests that 39% of Americans think that climate change is harming people in the US now (Leiserowitz et al., 2018). One important effect of instruction in the introductory biology course we examined was that, by the end of the semester, participants better understood that climate change is already harming people, with 63% correctly choosing “now” when asked to identify the point at which climate change would begin to harm people.

After instruction, participants not only began describing the harmful effects of climate change as more immediate, but also as more personal. When these students were asked how much they thought climate change would harm them personally, their most common response was “a moderate amount” (chosen by 43%) at the beginning of the semester, as compared to “a great deal” (chosen by 41%) at the end of the semester. Such a shift makes sense, considering that another instructional emphasis of the course was that climate change is linked to human health. In lecture, students were presented with this quotation from an editorial in a prominent medical journal: “Today, in the early part of the 21st century, it is critical to recognize that climate change poses the same threat to health as the lack of sanitation, clean water, and pollution did in the early 20th century” (Bauchner & Fontanarosa, 2014, p. 1519). Students were also presented with information on the myriad ways that climate change impacts human health, including via more frequent extreme heat and ozone exceedance days, increased vector‐borne diseases, greater food insecurity, and more natural disasters (Patz, Frumkin, Holloway, Vimont, & Haines, 2014). Given this content, students would have had ample cause to leave the course feeling that they were more likely to be personally harmed by a changing climate.

Returning to the quote at the beginning of this paper, the results of our study help us understand how climate change coursework might be informative, while also leading some students to report having an “anxiety attack.” Our results suggest that the quoted student is far from alone in being very worried about climate change: By the end of the semester, 58% of participants reported being very worried. In comparison, only 21% of Americans are “very worried” about climate change (Leiserowitz et al., 2018). Climate change can influence mental health, both as a result of climate‐related disasters and as a result of more gradual changes to climate (Trombley, Chalupka, & Anderko, 2017). Notably, climate change can elicit *solastalgia*: “the pain or distress caused by the loss of, or inability to derive, solace connected to the negatively perceived state of one's home environment” (Albrecht et al., 2007, p. S96; see also Albrecht, 2011). We hypothesize that the high level of worry expressed by our participants may result from them becoming more certain that climate change is happening and more aware of the immediacy of its impacts, while at the same time retaining pessimistic views about whether humans will actually act to reduce climate change. Importantly, and perhaps counter‐intuitively, coursework that focuses too narrowly on readerly climate literacy may create new barriers to writerly climate action.

What can educators do if they want students to leave their courses with an increased understanding of the science of climate change *and* with a sense of purpose and empowerment? As a place to start, we recommend educators ask themselves questions like the following, as they (re)plan and (re)design their courses:

1. *“What do my students already know or think about climate change as they enter my course?”* Because no student enters her introductory science course a blank slate, it is important for instructors to get a sense for student conceptions of climate change—both to aid in addressing misconceptions (see Monroe, Plate, Oxarart, Bowers, & Chaves, 2017; Pascua & Chang, 2015) and to ensure that valuable class time can be devoted to extending what students know, rather than attempting to convince them of things they come to us already accepting. Diagnostically or formatively assessing students' understandings of climate change—whether through surveying or testing them in some manner, as we did (see surveys in Appendices S2 and S3) and as others have done (e.g., Pascua & Chang, 2015; Versprille et al., 2017); or by engaging in additional activities, like concept mapping (Rebich & Gautier, 2005) and other in‐class writing tasks (see, e.g., Hand et al., 1999)—can provide a powerful basis for tailoring instruction to meet students where they are at, and to meet their intellectual and affective needs in ways that promote writerly climate literacy.
2. *“What things are being done locally (by individuals or communities) that are effectively combating climate change?”* Important as it may be to support students in understanding global climate systems, there is a need to complement this knowledge with a nuanced understanding of how effective climate action can be, and is being, taken at local scales. Focusing only on the problem of climate change can be, in more senses than one, a problematic approach to promoting climate literacy. To use the “bright spot” framing (Heath & Heath, 2010), instructors can ask themselves and their students: *What seems to be working in our local context, and how can we replicate, expand, or intensify this bright spot?* By focusing on local bright spots, instructors can help students reframe a large, very difficult problem so that it seems less distant and more actionable, perhaps increasing students' perceived self‐efficacy—the absence of which can be a significant barrier to climate intervention and behavioral change (see, e.g., Corner et al., 2015; Gifford, 2011).
3. *“What can we gain by fighting climate change?”* Relatedly, focusing on potential gains from tackling climate change—rather than fixating exclusively on what we lose if we do not—may position students to feel more positively toward taking action to address climate change (Corner et al., 2015; Gifford & Comeau, 2011). As Ojala (2012, 2015) contends, a “constructive” sense of hope can be a powerful source for climate engagement. This shift in framing can take the form of how we present information in our courses or in the work we ask students to complete. Asking students to internalize information about impending climate catastrophe, and testing them on this knowledge, may send a different, more disempowering message than the one communicated by asking them to think through what the world might look like—5, 10, or 50 years hence—if particular actions are taken to slow or reverse climate change. Meaningfully answering this latter kind of question requires students to understand the severity of climate change's projected impacts, but it does not take knowledge of climate catastrophe as its foregone conclusion. Instead, it invites students to imagine what it might look like to compose an alternative future.
4. *“How can course activities and assignments support students in thinking through their role(s) in climate change solutions (across spatial and temporal scales)?”* Because this last question intersects with and integrates insights from the preceding three, we discuss it at greater length. A 2017 systematic review of climate change education research found that pedagogically successful strategies often took the form of “activities that allow learners to actively engage with concepts, discuss their understanding, practice actions, and engage with relevant, local examples of climate change impacts” (Monroe et al., 2017, p. 15). Classroom activities and assignments can support students in revising, deepening, or extending their prior understandings in a writerly, socially conscious manner. By providing concrete opportunities for students to explore how human actions shape climate outcomes, our courses can help them develop a critical repertoire for appraising how individual and collective practices participate in climate change—and how action can be taken to slow and adapt to it.

For example, an assignment might ask students to focus on identifying solutions for real world scenarios, such as by leading the World Climate Simulation, which has been shown to leave participants with greater feelings of urgency and hope and a greater desire to act in response to climate change (Rooney‐Varga et al., 2018). Instructors might engage students in analyzing and writing responses to local or national climate‐related news coverage, or else follow the example Hand et al. (1999) set by tasking students to compose their own mock newspaper articles (pp. 1030–1031). Relatedly, another project could be to have students consider the potential benefits, limitations, and implications of the various interventions listed by Project Drawdown (Hawken, 2017; Project Drawdown, n.d.), and to brainstorm ways to publicize their conclusions for one or more specific audiences (e.g., climate‐skeptical relatives, local community members, state or national policy makers). In any of these examples, students could be asked to think through how the findings from these activities could be used to guide effective social and/or scientific intervention—positioning students to imagine what they learn in the classroom as a prelude to potential action in the world beyond it.
As part of this pedagogical process, it is important to bear in mind that climate change is a complexly anthropogenic problem—driven, experienced, and responded to by humans. For this reason, robust climate literacy requires attention to global climate justice and injustice: asking how structural injustices (e.g., classism, racism, colonialism, sexism) relate to—even exacerbate—climate change, documenting climate change's demographically disparate impacts, addressing issues of climate accountability and responsibility, and considering the ethical implications of climate‐related (in)action (see, e.g., Harlan et al., 2015; Lotz‐Sisitka, 2010; Schlosberg & Collins, 2014; Shue, 2014; Zeidler & Newton, 2017). One direct way for courses to draw in and draw on some of these themes is to discuss (and develop activities around) how demographic groups and geographic regions are asymmetrically implicated in and impacted by climate change, helping students to understand that “those countries”—and populations—“most at risk from the consequences of climate change are ironically and paradoxically also the ones that have contributed least to climate change” (Lotz‐Sisitka, 2010, p. 72; see also Cuomo, 2011; Whyte, 2014). In this vein, increased classroom attention to climate justice promises not only to foreground the ethical stakes and implications of climate change, but also to shift the curricular focus of introductory courses toward a more writerly climate literacy, attentive to the need and potential for intervention.

These four questions are in no way exhaustive, but we believe they provide a useful starting place for designing and enacting instruction that promotes the kind of writerly climate literacy necessary for translating and recomposing climate knowledge into climate action. Such promotion is necessary if we are to meet the challenges climate change poses, for as science education scholars like Kagawa and Selby (2010), González‐Gaudiano and Meira‐Cartea (2010), and Lotz‐Sisitka (2010) argue, scientific knowledge must be counterbalanced with active pedagogical attention to agency, ethics, and intervention.

The importance of shifting from focusing on a readerly literacy to a writerly one in introductory science course pedagogy is hard to overstate. Many students in introductory biology likely take the course because it is required (perhaps as preparatory coursework for health‐oriented careers), irrespective of whether they have a deep interest in climate, ecology, or the environment—affording instructors the opportunity to reach a group of students who might not typically think or learn about climate change, and to connect its impacts with their lives in ways that inspire them to act. Returning to the “preaching to the choir” component of our title, students in this kind of course may be in the proverbial choir, but not all of them are there entirely of their own volition.

We suspect that few science educators would argue that ecology majors are the only population responsible for responding to the effects of climate change. Climate justice is a project requiring collective action from professional scientists and nonscientists alike. Our introductory courses are one place for us to invite and inspire a broader population to take part in this work—and, in turn, to extend this invitation to the broader publics they participate in. To the extent that our courses can provide a foundation for future climate activism, how we teach about climate change is *in itself* a matter of climate justice. Learning about climate change means more than learning about climate systems—it means also learning about active ways to equitably, productively, and sustainably intervene in those systems. We argue that classroom instruction does a disservice to students if it deepens their knowledge about climate change but also leaves them feeling such despair and disempowerment that they are disinclined to try to make a difference. Our study suggests that instructors should go beyond simply characterizing the causes and consequences of climate change. To the extent we are preaching to the proverbial choir, we do well to empower students and support them in composing new verses. In increasing students' senses of possibility and efficacy, instructors can help them emerge more fully climate literate: ready not just for readerly recognition, but also writerly action.

## CONFLICT OF INTEREST

None declared.

## AUTHOR CONTRIBUTIONS

All authors were involved in the design of the study and the analysis of results. All authors contributed to the writing and editing of the manuscript.

## Data Availability

Data and code are available at https://doi.org/10.5281/zenodo.3334292
