# Supplementary figures and images for: Preaching to the choir or composing new verses? Toward a writerly climate literacy in introductory undergraduate biology
Source: Ecol Evol. 2019 Oct 28;9(22):12360–73. doi: 10.1002/ece3.5736 (PMC6876685; doi:10.1002/ece3.5736)

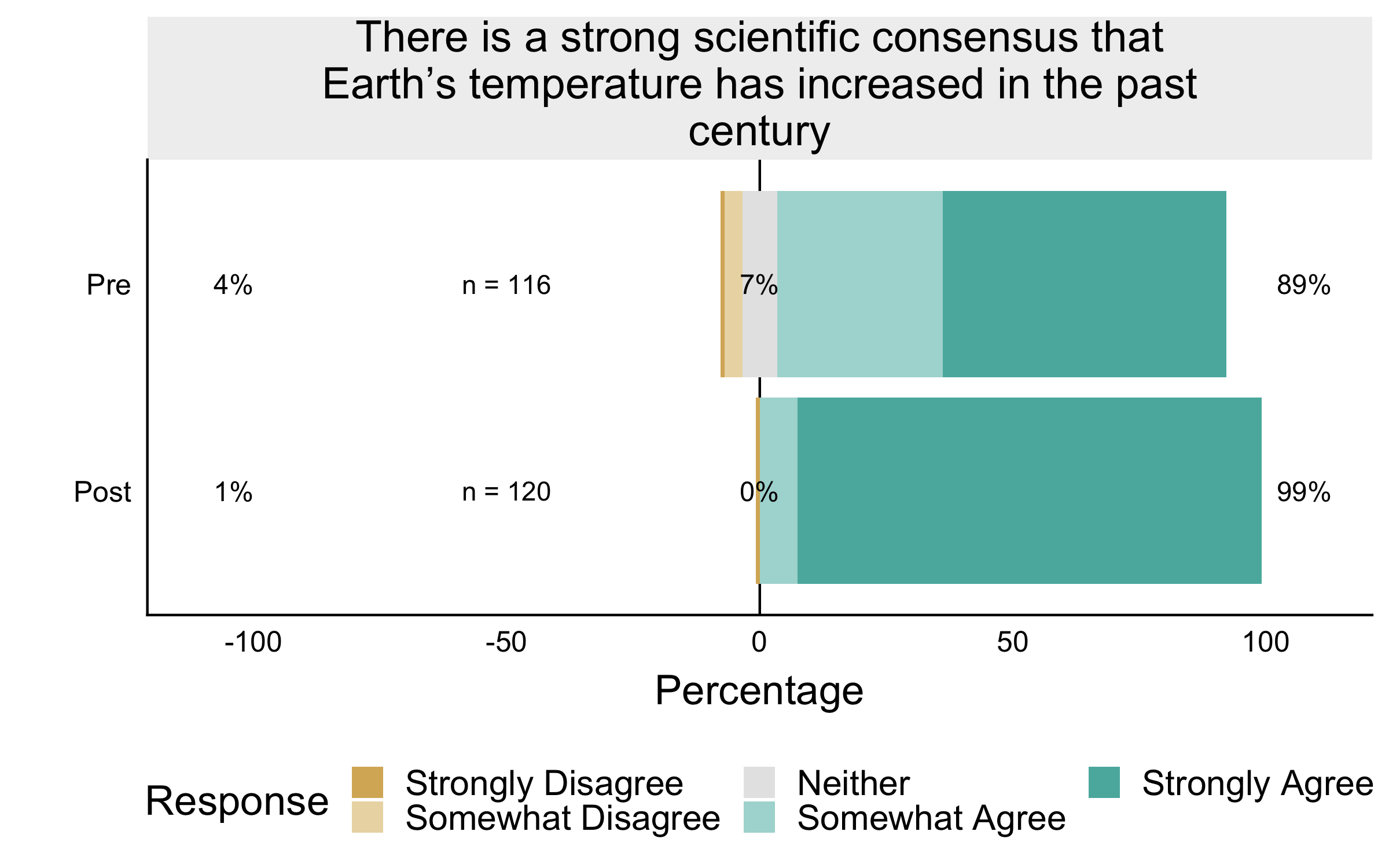

Supplement: Supplementary file 1 [file ECE3-9-12360-s001.tif]

**Could you/did you change your views? (n=120)**

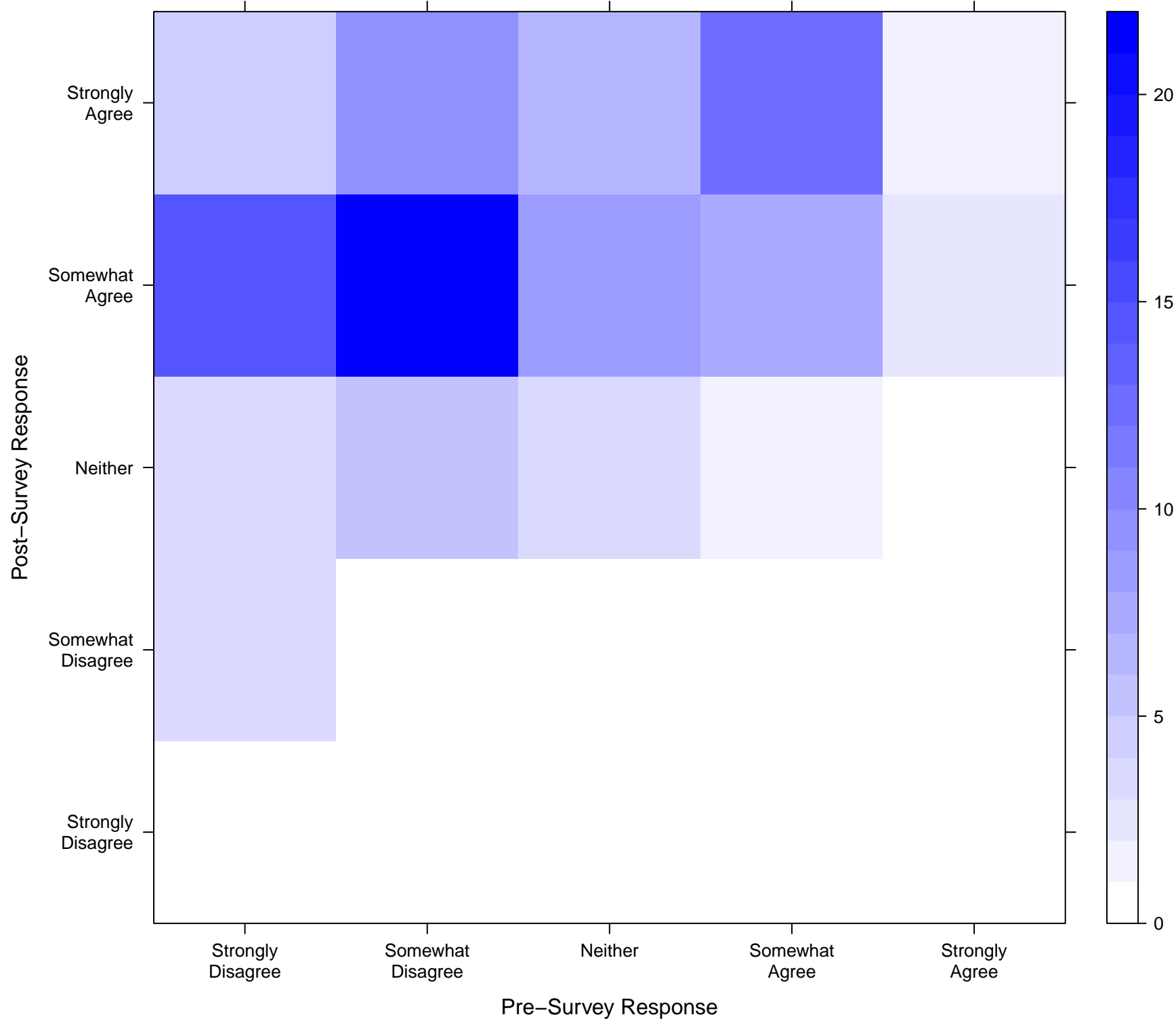

Supplement: Supplementary file 2 [file ECE3-9-12360-s002.pdf]

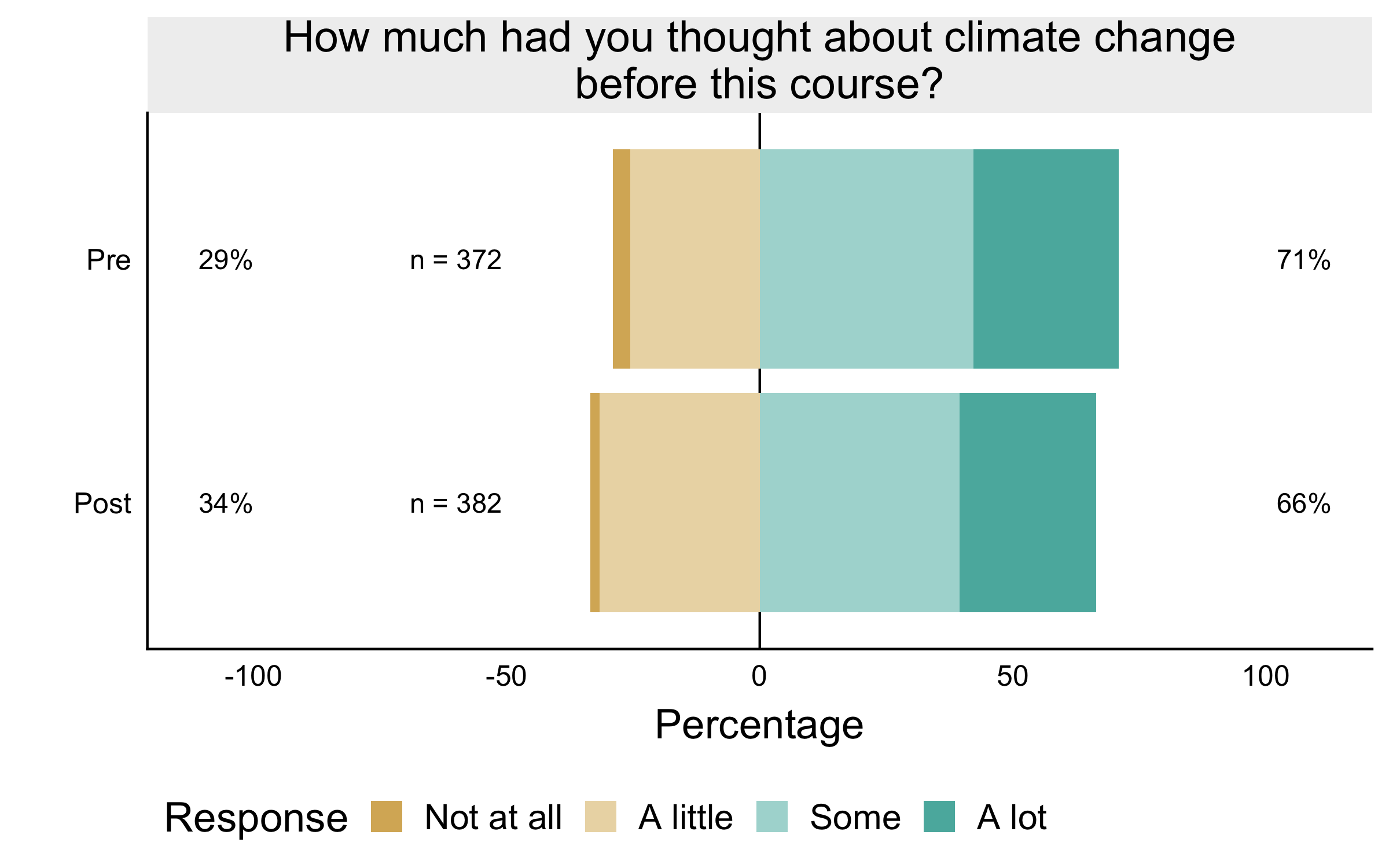

Supplement: Supplementary file 3 [file ECE3-9-12360-s003.tif]

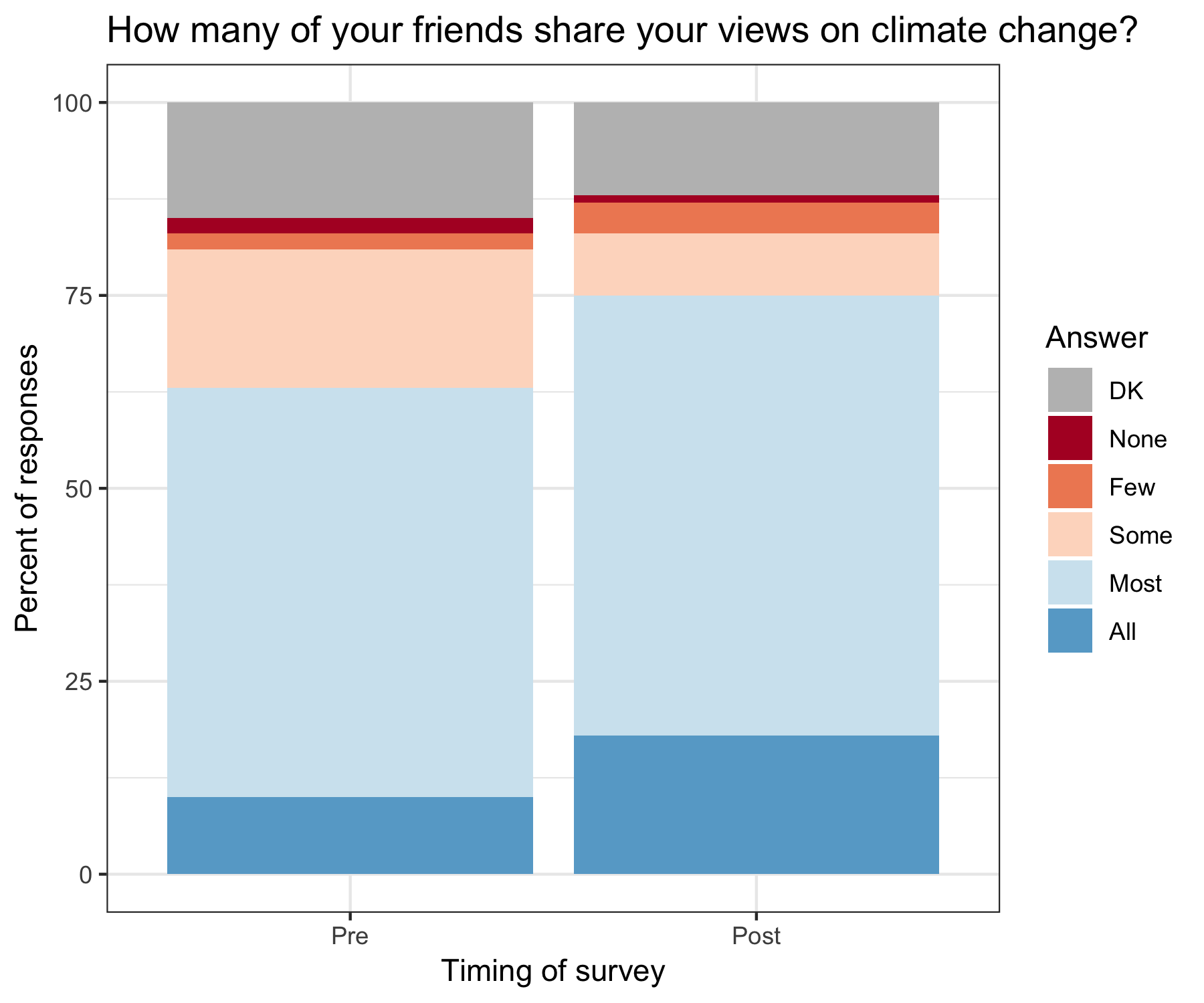

Supplement: Supplementary file 4 [file ECE3-9-12360-s004.tif]
