# Supplementary material for: Preaching to the choir or composing new verses? Toward a writerly climate literacy in introductory undergraduate biology
Source: Ecol Evol. 2019 Oct 28;9(22):12360–73. doi: 10.1002/ece3.5736 (PMC6876685; doi:10.1002/ece3.5736)
Supplement: Supplementary file 5 [file ECE3-9-12360-s005.docx]

**Appendix**

**Supplemental results**

*Do students recognize the strong scientific consensus that Earth’s temperature has increased in the past century?*

In 2017, nearly all participants recognized at the beginning of the semester that there is a strong scientific consensus that Earth’s temperature has increased in the past century. At the end of the semester, 7% “somewhat agreed” and 92% “strongly agreed” (Figure S1).

*Do students report the course as changing how they think about climate change?*

Combined across the two semesters, 68% of participants agreed (24% “strongly,” 44% “somewhat”) with the statement that “This course changed how I think about climate change,” versus 24% who chose “neither agree nor disagree” and 8% who disagreed (Figure S2).

*How easily do students think they could change their minds about climate change?*

In 2017, we asked students at the beginning of the course: “How much do you agree or disagree with the following statement? ‘I could easily change my mind about climate change.’” 65% of participants disagreed that they could easily change their minds. However, at the end of the semester, 79% agreed that the course had changed how they thought about climate change. The most common combination of responses was the one where participants chose “somewhat disagree” at the beginning of the semester and “somewhat agree” at the end of the semester (Figure S2). One possible explanation for this pattern is that these students interpreted the question at the beginning of the semester as asking whether they could easily change their minds regarding whether climate change is occurring.

*How much had students thought about climate change before the course?*

Participants’ responses in 2017 and 2018 to the question “How much had you thought about climate change before this course” were largely consistent at the beginning and end of the semester, though there was a trend toward these students saying at the end of the course that they had thought less about climate change prior to the course (Figure S3). The number of participants who said they had only thought “a little” about climate change before the course increased marginally over the course of the semester (𝜒2 = 3.18, p = 0.075; 26% on the pre-survey vs. 32% on the post).

*How many friends share your views on climate change?*

Most participants reported that most of their friends shared their views on climate change, and those responses were generally consistent at the beginning and end of the semester (53% chose “most” at the beginning of the semester vs. 57% at the end; Figure S4).
