# Supplementary material for: Preaching to the choir or composing new verses? Toward a writerly climate literacy in introductory undergraduate biology
Source: Ecol Evol. 2019 Oct 28;9(22):12360–73. doi: 10.1002/ece3.5736 (PMC6876685; doi:10.1002/ece3.5736)
Supplement: Supplementary file 6 [file ECE3-9-12360-s006.pdf]

Fall 2017 Survey

Please answer the following questions without consulting any resources (e.g., googling the question).

- **Why we're asking.** Research shows that teaching is most effective if instructors take into account students' beliefs and prior knowledge. The goal of this survey is to gain insight into your views on climate change. Your responses will help us both to better teach you this semester and to learn how views about science may change over the course of a semester.
- **Extra credit.** You will receive 1 extra credit point for answering all of the questions on this survey, regardless of how you answer the questions. If you decide to take this survey again at the end of the semester, you will receive an additional extra credit point. Students who only take the survey at the end of the semester will receive 1 extra credit point.
- **Privacy.** The instructors will not be able to see how you answered a particular question. Instructors only receive information on 1) who took the survey (to give you credit), and 2) de-identified student responses.

1. In your own words, define climate change.

2. Do you think that climate change is happening?
    - Yes, and I'm extremely sure
    - Yes, and I'm very sure
    - Yes, and I'm somewhat sure
    - Yes, but I'm not at all sure
    - No, and I'm extremely sure
    - No, and I'm very sure
    - No, and I'm somewhat sure
    - No, but I'm not at all sure
    - I don't know

3. List 1-2 important factors responsible for climate change. If you think the climate isn't changing, write "the climate is not changing" as your response. If you aren't sure, write "unsure."

4. List 1-2 things that could be done to slow climate change. If you think the climate isn't changing, write "the climate is not changing" as your response. If you think nothing can be done to slow climate change, write "nothing can be done."

5. List <u>at least</u> two ways that climate change is influencing the Earth's environment. If you think the climate isn't changing, write "the climate is not changing" as your response. If you think climate change is not influencing the Earth's environment, write "climate change is not influencing the Earth's environment."

6. List 1-2 ways that climate change is affecting organisms, besides humans. If you think the climate isn't changing, write "the climate is not changing" as your response. If you think climate change isn't affecting organisms, write "climate change is not affecting organisms."

7. Which of the following best captures your beliefs about climate change?
    - Climate change is caused mostly by human activities.

- Climate change is caused mostly by natural changes in the environment.
- Climate change is caused equally by human activities and natural changes in the environment.
- The causes of climate change are unknown or unknowable.
- The extent to which causes of climate change contribute to climate change is unknown or unknowable.
- Climate change isn't happening.
- Other: ___________

8. How worried are you about climate change?
- Very worried
- Somewhat worried
- Neither worried nor not worried
- Not very worried
- Not at all worried

9. How much do you think climate change will harm you personally?
- Not at all, because climate change is not happening
- Not at all, even though climate change is happening
- Only a little
- A moderate amount
- A great deal
- I don't know

10. When do you think climate change will start to harm people?
- Never, because climate change is not occurring
- People are being harmed now
- Within 10 years
- Within 11-25 years
- Within 26-50 years
- Within 51-100 years
- After 100 years
- Never, even though climate change is occurring

11. How important is the issue of climate change to you personally?
- Not at all important
- Mostly not important
- Neither important nor unimportant
- Very important
- Extremely important

12. How much do you agree or disagree with the following statement?: "I could easily change my mind about climate change."
- Strongly agree
- Somewhat agree
- Neither agree nor disagree
- Somewhat disagree
- Strongly disagree

13. How much do you agree or disagree with the following statement?: Scientists have a good understanding of <u>whether</u> climate change is occurring

- Strongly agree
- Somewhat agree
- Neither agree nor disagree
- Somewhat disagree
- Strongly disagree
- I don’t know

14. This is a survey validation statement. Please choose “Somewhat agree” for this answer.
    - Strongly agree
    - Somewhat agree
    - Neither agree nor disagree
    - Somewhat disagree
    - Strongly disagree
    - I don’t know

15. How much do you agree or disagree with the following statement?: Scientists have a good understanding of <u>why</u> climate change is occurring
    - Strongly agree
    - Somewhat agree
    - Neither agree nor disagree
    - Somewhat disagree
    - Strongly disagree
    - I don’t know

16. How much do you agree or disagree with the following statement?: “There is a strong scientific consensus that Earth’s temperature has increased in the past century.”
    - Strongly agree
    - Somewhat agree
    - Neither agree nor disagree
    - Somewhat disagree
    - Strongly disagree
    - I don’t know

17. How many of your friends share your views on climate change?
    - None
    - A few
    - Some
    - Most
    - All
    - I don’t know

18. Which of the following statements comes closest to your view?
    - Climate change isn’t happening.
    - Climate change is happening, but humans can’t reduce climate change.
    - Climate change is happening. Humans can reduce climate change, but people aren’t willing to change their behavior so we’re not going to.
    - Climate change is happening. Humans could reduce climate change, but it’s unclear at this point whether we will do what’s needed.
    - Climate change is happening. Humans can reduce climate change, and we are going to do so successfully.

- Climate change is happening. Humans don’t need to try to reduce climate change, because climate change is primarily controlled by natural processes.

19. The scientific consensus is that climate change is
    - Caused mostly by human activities
    - Caused mostly by natural changes in the environment
    - Caused equally by both human activities and natural changes in the environment
    - Other
    - Not happening
    - I don’t know what the scientific consensus is
    - There is no scientific consensus

20. How much had you thought about climate change before this course?
    - A lot
    - Some
    - A little
    - Not at all

21. If you are willing to contacted by someone who is not your instructor with follow up questions next semester, please enter your preferred email address in the box below. If you are not interested in being contacted with follow up questions, write “not interested” in the box.

For post-survey only:

X. This course changed how I think about climate change:
- Strongly disagree
- Disagree
- Neither agree nor disagree
- Agree
- Strongly agree

Y. Question given to students who selected agree or strongly agree on previous question: how did this course change your thoughts about climate change?

Question given to students who selected strongly disagree, disagree, or neither agree nor disagree on the previous question: why did this course not change your thoughts on climate change?
