# Supplementary material for: Preaching to the choir or composing new verses? Toward a writerly climate literacy in introductory undergraduate biology
Source: Ecol Evol. 2019 Oct 28;9(22):12360–73. doi: 10.1002/ece3.5736 (PMC6876685; doi:10.1002/ece3.5736)
Supplement: Supplementary file 7 [file ECE3-9-12360-s007.pdf]

Winter 2018 Survey

Please answer the following questions without consulting any resources (e.g., googling the question).

- **Why we're asking.** Research shows that teaching is most effective if instructors take into account students' beliefs and prior knowledge. The goal of this survey is to gain insight into your views on climate change. Your responses will help us both to better teach you this semester and to learn how views about science may change over the course of a semester.
- **Extra credit.** You will receive 1 extra credit point for answering all of the questions on this survey, regardless of how you answer the questions. If you decide to take this survey again at the end of the semester, you will receive an additional extra credit point. Students who only take the survey at the end of the semester will receive 1 extra credit point.
- **Privacy.** The instructors will not be able to see how you answered a particular question. Instructors only receive information on 1) who took the survey (to give you credit), and 2) de-identified student responses.

1. In your own words, define climate change.

2. Do you think that climate change is happening?
   - Yes, and I'm extremely sure
   - Yes, and I'm very sure
   - Yes, and I'm somewhat sure
   - Yes, but I'm not at all sure
   - No, and I'm extremely sure
   - No, and I'm very sure
   - No, and I'm somewhat sure
   - No, but I'm not at all sure
   - I don't know

3. I think that climate change is:
   - Not happening
   - Caused *only* by natural activities
   - Caused *only* by natural changes in the environment
   - Caused *mostly* by human activities, but also by natural activities
   - Caused *mostly* by natural changes in the environment, but also by human activities
   - Caused *equally* by both human activities and natural changes in the environment
   - has causes that are unknown or unknowable.
   - Other

4. List 1 action that could be taken to slow climate change. If you think the climate isn't changing, write "the climate is not changing" as your response. If you think nothing can be done to slow climate change, write "nothing can be done."

5. List 1 way that climate change is affecting organisms besides humans. If you think the climate isn't changing, write "the climate is not changing" as your response. If you think climate change isn't affecting organisms, write "climate change is not affecting organisms."

6. List 1 way that climate change is affecting humans. If you think the climate isn't changing, write "the climate is not changing" as your response. If you think climate change isn't affecting humans, write "climate change is not affecting humans."

7. How worried are you about climate change?
   - Very worried
   - Somewhat worried
   - Neither worried nor not worried
   - Not very worried
   - Not at all worried

8. Why are you worried about climate change? {only given to students who selected "somewhat worried" or "very worried" on previous question}

9. How much do you agree or disagree with the following statement: Scientists have a good understanding of <u>whether</u> climate change is occurring
   - Strongly agree
   - Somewhat agree
   - Neither agree nor disagree
   - Somewhat disagree
   - Strongly disagree
   - I don't know

10. How much do you agree or disagree with the following statement: Scientists have a good understanding of <u>why</u> climate change is occurring
    - Strongly agree
    - Somewhat agree
    - Neither agree nor disagree
    - Somewhat disagree
    - Strongly disagree
    - I don't know

X. This is a survey validation statement. Please choose "Somewhat agree" for this answer.
   - Strongly agree
   - Somewhat agree
   - Neither agree nor disagree
   - Somewhat disagree
   - Strongly disagree
   - I don't know

{note: this survey validation question was inadvertently omitted from the pre-survey}

11. The scientific consensus is that climate change is
    - Caused mostly by human activities
    - Caused mostly by natural changes in the environment
    - Caused equally by both human activities and natural changes in the environment
    - Other
    - Not happening

- I don’t know what the scientific consensus is
- There is no scientific consensus

12. How much had you thought about climate change before this course?
    - A lot
    - Some
    - A little
    - Not at all

13. If you are willing to contacted by someone who is not your instructor with follow up questions next semester, please enter your preferred email address in the box below. If you are not interested in being contacted with follow up questions, write “not interested” in the box.

For post-survey only:
X. This course changed how I think about climate change:
- Strongly disagree
- Disagree
- Neither agree nor disagree
- Agree
- Strongly agree

Y. Question given to students who selected agree or strongly agree on previous question: how did this course change your thoughts about climate change?
Question given to students who selected strongly disagree, disagree, or neither agree nor disagree on the previous question: why did this course not change your thoughts on climate change?
