# Supplementary material for: Preaching to the choir or composing new verses? Toward a writerly climate literacy in introductory undergraduate biology
Source: Ecol Evol. 2019 Oct 28;9(22):12360–73. doi: 10.1002/ece3.5736 (PMC6876685; doi:10.1002/ece3.5736)
Supplement: Supplementary file 8 [file ECE3-9-12360-s008.docx]

Figure S1. Student Likert-scale responses in 2017 to the statement: “There is a strong scientific consensus that Earth’s temperature has increased in the past century.” Nearly all participants recognized that there is a strong scientific consensus that Earth’s temperature has increased in the past century, and they shifted to thinking this more strongly by the end of the semester. This figure omits the 4 students who chose “I don’t know” on the beginning of semester survey; none chose that option at the end of the semester.

Figure S2. At the beginning of the 2017 semester, most participants disagreed that they could easily change their minds about climate change, but at the end of the semester, agreed that the course had changed how they think. On the presurvey, students were asked how much they agreed or disagreed with the following statement: “I could easily change my mind about climate change.” On the postsurvey, students were presented with the statement “This course changed how I think about climate change” and then were asked to choose one of the responses shown on the y-axis. The color in each square represents the number of participants who answered with a specific presurvey and postsurvey response pairing. For example, participants who strongly disagreed with “I could easily change my mind about climate change” at the beginning of the semester and who strongly agreed with “This course changed how I think about climate change” are represented in the upper left hand box of the figure, and the light blue shading indicates 5 students gave that pair of responses. The most common pair of responses was “somewhat disagree” when asked on the presurvey if they could change their minds about climate change and “somewhat agree” when asked on the postsurvey if the course changed how they think about climate change; 22 students chose this pair of responses.

Figure S3. When asked, “How much had you thought about climate change before this course?” participants gave generally consistent responses at the beginning and end of the semester, but with a trend toward reporting at the end of the course that they had thought less about climate change prior to the course.

Figure S4. Participants’ responses in 2017 to the question “How many of your friends share your views on climate change?” were largely consistent at the beginning and end of the semester, with most of these students saying “most” friends share their views at the beginning and end. Note: “DK” = “I don’t know.”
